# Embracing complexity in *Drosophila* cancer models

**DOI:** 10.1242/dmm.049513

**Published:** 2022-03-28

**Authors:** Courtney Choutka, Cecilia Cabrera, Susumu Hirabayashi

**Affiliations:** 1 Medical Research Council London Institute of Medical Sciences, Du Cane Road, London W12 0NN, United Kingdom; 2 Institute of Clinical Sciences, Faculty of Medicine, Imperial College London, Du Cane Road, London W12 0NN, United Kingdom

**Keywords:** Ageing, Cachexia, Cancer, *Drosophila*, Metastasis, Multimorbidity, Obesity, Therapeutics, Tumour diversity

## Abstract

Cancer continues to be a leading cause of death worldwide, largely due to metastases and cachexia. It is a complex disease that is commonly associated with a variety of comorbidities. With global increases in ageing populations and obesity, multimorbidity is a rapidly growing clinical issue in the context of cancer. Cancer is also genetically heterogeneous, with a tumour's unique profile determining its incidence of metastasis, degree of cachexia and response to therapeutics. These complexities of human cancer are difficult to replicate in animal models and are, in part, responsible for the failures in translational cancer research. In this Perspective, we highlight the fruit fly, *Drosophila melanogaster*, as a powerful model organism to investigate multimorbidity and tumour diversity. We also highlight how harnessing these complexities in *Drosophila* can, potentially, enhance cancer research and advance therapeutic discoveries.

## Abstract (precis)

**Summary:** Comorbidities and tumour genetic diversity more accurately reflect the cancer patient landscape but are largely neglected in animal models. *Drosophila* holds the potential to address these complexities to better understand their impacts on cancer development.

## Introduction

Cancer is a systemic disease that can affect several host tissues by metastasising to distant organs or by inducing the muscle wasting syndrome cachexia (Biswas and Acharyya, 2020). Furthermore, systemic metabolic conditions, such as obesity, promote the risk and progression of various cancers (Calle and Kaaks, 2004; Lauby-Secretan et al., 2016; Renehan et al., 2008). Over recent decades, the fruit fly *Drosophila melanogaster* has been increasingly used to model and study both cancer (Chatterjee and Deng, 2019; Gonzalez, 2013; Sonoshita and Cagan, 2017) and metabolic diseases (Chatterjee and Perrimon, 2021; Kim et al., 2021). More recently, *Drosophila* has emerged as a tool for studying the obesity–cancer association (Hirabayashi, 2016; Warr et al., 2018), and cancer cachexia (Figueroa-Clarevega and Bilder, 2015; Kwon et al., 2015; Liu et al., 2022). With increased interest in using flies to study host–tumour interactions (Bilder et al., 2021; Wong and Verheyen, 2021), this is a timely opportunity to consider how the fly research community can further embrace the potential of *Drosophila* cancer research.

In this article, we identify two major challenges in animal-based cancer research that can be addressed by leveraging the strengths of *Drosophila* models: multimorbidity and tumour diversity. Multimorbidity – the coexistence of multiple chronic conditions – is becoming increasingly prevalent with larger aged populations and longer life expectancies (Violan et al., 2014). The incidence of cancer increases with age, possibly due to increasing genomic instability, changes in the endocrine system and decline in tissue function (de Magalhães, 2013). Age-associated disorders can also coexist with cancer (Pamoukdjian et al., 2018). For example, sarcopenia – a decrease in skeletal muscle mass and function – occurs in up to 10% of individuals over 65 years of age (Demontis et al., 2013; Morley et al., 2014; Dhont et al. 2021) and is associated with increased cancer mortality (Au et al., 2021). Obesity also increases the risk of various cancers (Calle and Kaaks, 2004; Lauby-Secretan et al., 2016; Renehan et al., 2008), and promotes malignant cancer progression (Annett et al., 2020). In addition, obesity and age-associated organ decline can negatively affect surgical outcomes in cancer treatment (Baracos and Arribas, 2018; Slawinski et al., 2020). The interpatient tumour heterogeneity adds another layer of complexity to cancer research. Our mechanistic understanding of how the genetic profiles of tumours are associated with obesity and ageing is limited. Here, we consider how addressing obesity, ageing and tumour diversity in *Drosophila* cancer models is a feasible approach to driving fundamental discovery with the ultimate aim of improving translational cancer research.

## Challenges in animal cancer models: multimorbidity and inter-patient tumour diversity

Multimorbidity is a growing health concern that is commonly associated with cancer patients. However, the effects of comorbidities and ageing have been overlooked in current animal models of cancer mostly owing to issues with timing the experiments – as a typical mouse model takes 16-20 weeks to develop diet-induced obesity (Wang and Liao, 2012) and the mouse is considered ‘old’ at 18-24 months of age. Aged cancer mouse models also create an ethical dilemma regarding animal welfare: to study late-stage cancers, animals would be exposed to severe phenotypes of decline.

Cancer is a highly heterogeneous disease; although tumours can emerge from similar tissues, their genetic signatures differ from patient to patient. Currently, typical animal cancer models involve mouse studies using a ‘one tumour, one model’ approach, with discovered mechanisms or treatments not being assessed with genetically distinct tumours or in the presence of other variables, such as variations regarding age and diet (Gengenbacher et al., 2017). Analysing multiple tumour models would validate findings that are common to different tumours and reveal important tumour-specific differences. In addition, animal studies on tumour diversity and therapeutic response could provide valuable information for patient stratification, and influence the design of future clinical trials. Unfortunately, comparative analyses of multigenic tumour models are difficult to achieve in mouse studies, mainly owing to the time, effort and cost involved in generating, maintaining and analysing multiple tumour models in parallel.

In the following sections, we illustrate how *Drosophila* cancer models can allow us to address the complexities outlined above, and propose how they could further advance cancer research.

## Embracing multimorbidity

**Fig. 1.**
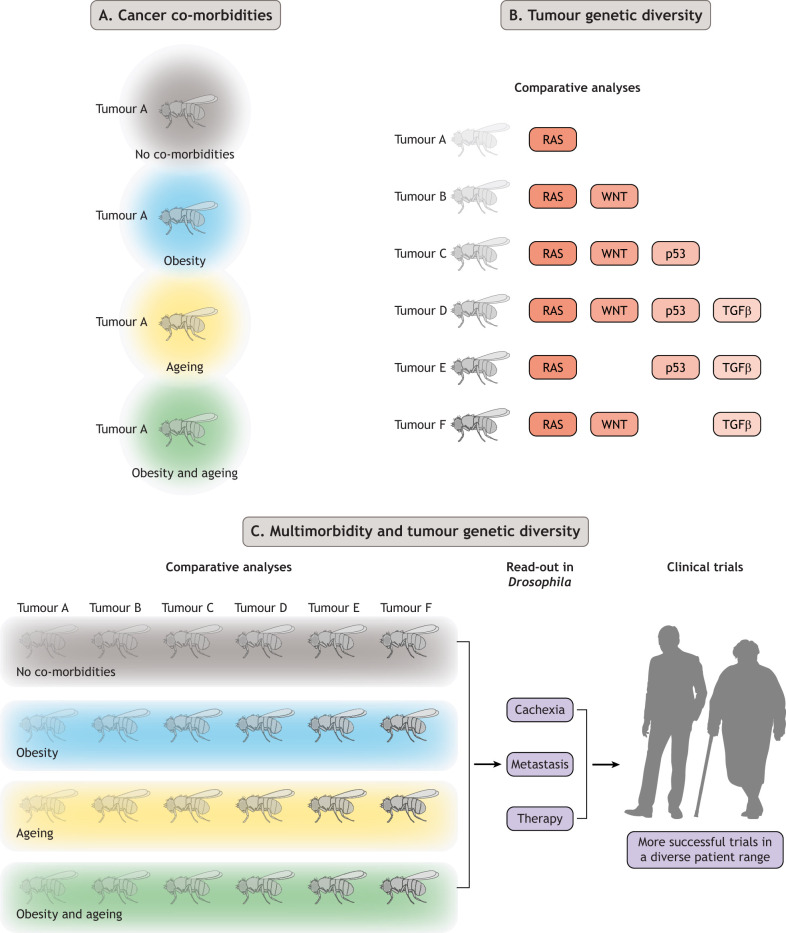
***Drosophila melanogaster* as a cancer model for addressing multimorbidity and inter-patient tumour diversity.** (A) Without altering genetics, *Drosophila* can be used to model cancer in combination with other co-morbidities, such as obesity and ageing, for a more accurate patient simulation. (B) Comparative analysis can be carried out on a plenitude of genetic combinations targeting commonly dysregulated pathways (such as RAS, WNT, p53 and TGFβ) to compare patients' unique tumours. This can also be analysed in different tissues to understand the importance of tumour location. (C) The true power of *Drosophila* lies in the ability to simultaneously study the complexities of multimorbidity and tumour diversity, as well as their effects on cachexia, metastasis and therapy. This can translate to more personalised and stratified approaches in treating patients and to improved success in clinical trials.

Modelling multimorbidity in the context of cancer has already been achieved through diet modification. *Drosophila* expedites this research significantly since a diet-induced obese phenotype can be achieved in one to three weeks (Birse et al., 2010; Musselman et al., 2011; Na et al., 2013; Skorupa et al., 2008). *Drosophila* that are fed diets high in fat or sugar display fat accumulation and insulin resistance (Musselman et al., 2011; Skorupa et al., 2008; Woodcock et al., 2015), with associated cardiac (Birse et al., 2010; Diop et al., 2015; Na et al., 2013) and renal dysfunction (Na et al., 2015; van Dam et al., 2020): traits that are similar to those acquired in human obesity and type II diabetes. A *Drosophila* larval model of tumours in the context of high sugar-induced obesity has provided mechanistic insights into how obesity can promote tumour malignancy (Hirabayashi et al., 2013; Hirabayashi and Cagan, 2015) and cachexia-like muscle wasting (Newton et al., 2020). In another larval model, a high-sugar diet promoted hyperplastic-to-neoplastic transformation of tumours requiring lactate dehydrogenase, a crucial enzyme of aerobic glycolysis (Eichenlaub et al., 2018). A different study in adult *Drosophila* demonstrated that a high-sugar diet enhanced tumour-like proliferation via a functional hexosamine biosynthetic pathway (Wong et al., 2020). These findings highlight the impact obesity can have on different tumours. Future discoveries, in the context of obesity and cancer, can be taken advantage of in existing *Drosophila* models of cancer dissemination, metastasis and cachexia (Bangi et al., 2016; Campbell et al., 2019; Liu et al., 2022) (Fig. 1A). A straightforward approach to understanding how and if tumours respond to obesity could be achieved by simply altering the diet in established cancer models. In addition, one could assess how tumours respond to the microbiome by altering the diet (Grenier and Leulier, 2020; Douglas, 2018). Collectively, this research calls into question whether tumourigenesis can be impacted by other common human conditions.

**Figure:**
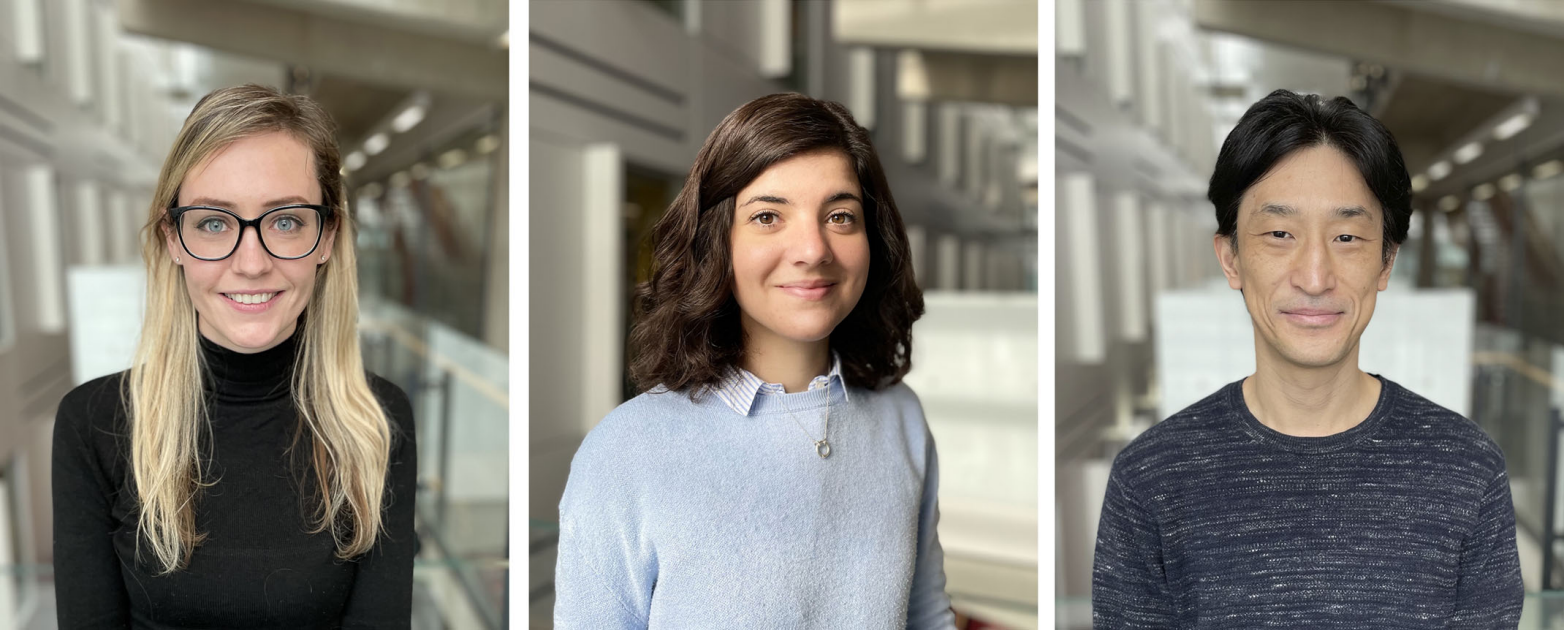
Courtney Choutka, Cecilia Cabrera and Susumu Hirabayashi

*Drosophila* is also a well-established model for studying ageing (Piper and Partridge, 2018) and age-associated diseases, including sarcopenia (Demontis and Perrimon, 2010; Miller et al., 2008; Owusu-Ansah et al., 2013; Rhodenizer et al., 2008). The relatively short lifespan of *Drosophila* adults (2-3 months) makes it feasible to use these animals to study the impact of ageing on cancer. Using the TARGET system (McGuire et al., 2004), a genetic tool that allows spatio-temporal control of gene expression, tumours can be induced at different ages to assess how tumour properties – such as dissemination, metastasis, cachexia and treatment response – can change in relation to age. In addition, altering the diet can address even more complex questions as to how cancers respond to both obesity and ageing simultaneously; thus achieving a more accurate patient simulation (Fig. 1A).

## Harnessing the genetic diversity of tumours and multimorbidity

The inter-patient heterogeneity of cancer is a challenging question to address in many animal cancer models. *Drosophila* models overcome this with their ability to efficiently produce and analyse large numbers of cohorts simultaneously. A recent study that established a repertoire of *Drosophila* colorectal cancer models is a salient example of such an approach (Bangi et al., 2016). Here, the authors analysed 32 different multigenic hindgut tumour models and demonstrated that different tumours exhibit (i) different invasive properties and (ii) different susceptibilities to drugs (Bangi et al., 2016). Another study demonstrated that genetically distinct tumours in the adult fly midgut impact organ wasting differently (Lee et al., 2021). This level of comparative analysis amongst cancers induced in the same tissue highlights an unparalleled strength of *Drosophila* models. By using similar comparative approaches, future studies can examine other tumour properties, such as the incidence and severity of tumour dissemination, metastasis and cachexia (Fig. 1B). Furthermore, it would be of interest to study whether the tissue of origin impacts the properties of tumours comprising various genotypes. These comparative approaches will help to improve our understanding of how genetic profile and tumour location affects cancer metastasis and cachexia.

“Although epidemiological studies have demonstrated that obesity promotes the risk and progression of cancers […] recent studies are beginning to reveal that genetically distinct tumours respond uniquely to obesity.”

Although epidemiological studies have demonstrated that obesity promotes the risk and progression of cancers originating from various tissues, such as colorectal cancers and breast cancers (Lauby-Secretan et al., 2016), recent studies are beginning to reveal that genetically distinct tumours respond uniquely to obesity. Reports indicate that the tumour-promoting effect of obesity depends on the tumour's genetic profile in prostate cancer (Pettersson et al., 2013), breast cancers (Neuhouser et al., 2015) and colorectal cancers (Campbell et al., 2010; Morikawa et al., 2013). Thus, we propose to combine *Drosophila* models in order to investigate how genetically diverse tumours are affected by obesity, ageing or both (Fig. 1C).

“[…] we believe that incorporating the patient's comorbidities in the fly ‘avatar’ models could improve the predictive power of pre-clinical animal models of cancer.”

## Modelling of personalised cancer therapy

The success rate of cancer clinical trials remains low: the approval rate for oncology therapeutics that entered Phase I clinical trials between 2011 and 2020 is 5.3%, just under half of that for non-oncological treatments (Thomas et al., 2021). In particular, treatment options for metastatic cancer and cachexia are limited (Tomasin et al., 2019). The predictive power of pre-clinical animal models can be improved by embracing the complexity of tumours as outlined in the previous sections. As mentioned earlier, a recent study used multiple multigenic *Drosophila* colorectal cancer models to identify the mechanism of emergence of drug resistance (Bangi et al., 2016). Furthermore, by leveraging the advances in personalised cancer genomic sequencing, attempts have been made to generate transgenic personalised fly ‘avatar’ models that carry unique combinations of genetic alterations found in cancer patients diagnosed with colorectal cancer or adenoid cystic carcinoma (Bangi et al., 2019, 2021). These fly ‘avatar’ models were then subjected to whole-animal cancer drug screening to inform personalised therapy recommendations (Bangi et al., 2019, 2021). These *Drosophila*-based approaches provide new avenues for developing personalised medicine. Given that pre-existing conditions, such as obesity, sarcopenia and cachexia, can affect cancer patient outcomes, treatment options and therapeutic response (Chowdhry and Chowdhry, 2019; Davis and Panikkar, 2019; Ross et al., 2019), we believe that incorporating the patient's comorbidities in the fly ‘avatar’ models could improve the predictive power of pre-clinical animal models of cancer.

## Conclusions

In this perspective article, we have discussed how *Drosophila* models can be used to address some of the current pressing challenges in modelling cancer. Rather than compare the advantages and disadvantages of different animal models of cancer, we encourage the reader to consider which questions could be more aptly answered using *Drosophila* models. We suggest that *Drosophila* models can complement existing animal models of cancer by responding to unmet needs in modelling complexity found in multimorbidity and tumour diversity. The insights gained may then be used to examine more-focused questions in mammalian models and human samples. *Drosophila* approaches may also help to partially replace, reduce and refine the use of mammalian animal models for cancer research, including personalised approaches, and its accompanying ethical quandaries. Altogether, *Drosophila* holds the potential to emulate the conditions and genetics of each patient to better understand their unique susceptibility to cachexia, metastasis or therapy.
